# Photodynamic therapy combined with cryotherapy for the treatment of nodular basal cell carcinoma

**DOI:** 10.3892/ol.2013.1504

**Published:** 2013-07-31

**Authors:** SUNG AE KIM, KYU SUK LEE, JAE-WE CHO

**Affiliations:** Department of Dermatology, Keimyung University School of Medicine, Dongsan-dong, Jung-gu, Daegu 700-712, Republic of Korea

**Keywords:** basal cell carcinoma, photodynamic therapy, carbon dioxide laser, cryotherapy

## Abstract

Carbon dioxide (CO_2_) laser ablation in combination with photodynamic therapy (PDT) has previously been successfully used to treat superficial basal cell carcinoma (BCC). However, the efficacy of this treatment modality is limited in the treatment of deeper lesions and the more aggressive subtypes of BCC. In order to improve the outcome of PDT, 8 BCC lesions of variable depths (4 lesions ≤2 mm and 4 lesions >2 mm) and subtypes (1 superficial, 6 nodular and 1 infiltrative) were treated with CO_2_ laser ablation in combination with PDT, followed by modified cryotherapy. The mean number of treatment sessions was 1.5 and the follow-up period was 22 months. All of the patients demonstrated a complete response and no recurrence of disease, while the majority of patients were satisfied with the cosmetic results upon follow-up examination. The combination therapy of CO_2_ laser ablation with PDT followed by modified cryotherapy demonstrated a good efficacy and satisfactory cosmetic outcomes in the treatment of nodular BCC.

## Introduction

The incidence of basal cell carcinoma (BCC) is increasing worldwide. As a result, the demand for novel and effective treatment modalities for BCC is high (1). BCC is commonly treated with surgical excision, curettage, carbon dioxide (CO_2)_ laser ablation or cryotherapy, depending on the tumor depth and the histological subtype of the BCC. Photodynamic therapy (PDT) has been widely used for the treatment of superficial BCC in preference to excision due to its minimal invasiveness and satisfactory cosmetic results (2). However, the efficacy of this treatment modality is limited in the treatment of deeper lesions and the more aggressive subtypes of BCC (3). Retreatment and recurrences of the disease are frequent if the tumor depth is >2 mm. Therefore, in order to improve the outcomes of BCC treatment, the present study attempted to combine CO_2_ laser ablation with topical methyl aminolevulinate (MAL) PDT and modified cryotherapy for the treatment of variable BCC.

## Materials and methods

### Patients

In total, 8 patients (2 males and 6 females) with a histological diagnosis of BCC (Table I) were admitted to the Department of Dermatology, Keimyung University Dongsan Hospital (Daegu, Republic of Korea) and included in the present study. Written informed consent was obtained from each patient. This study was approved by the Institutional Review Board of Dongsan Medical Center (Daegu, Korea).

### Treatment methods

**Figure 1:**
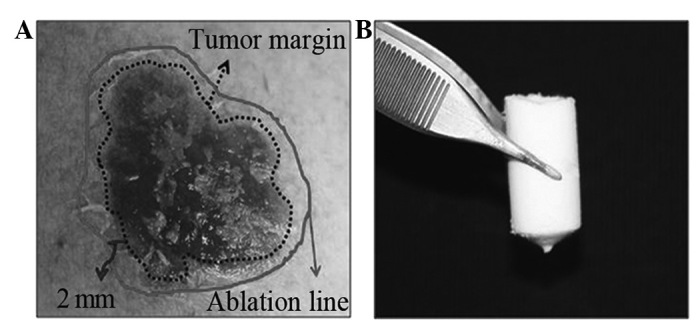
(A) Schematic figure depicting CO_2_ laser ablation along the tumor border and MAL application within a 2-mm tumor margin. (B) A cryostick constructed from solid carbon dioxide was applied to the lesion twice for 5 sec each time. MAL, methyl aminolevulinate; CO_2_, carbon dioxide.

Following CO_2_ laser ablation with a 2 mm margin (Fig. 1A), MAL (Metvix, Galderma, Oslo, Norway) was applied to a thickness of 1 mm covering the 2 mm of surrounding skin for 3 h. The lesion was then illuminated with light-emitting diodes (LEDs; Aktilite Lamp, Photocure ASA, Oslo, Norway) at a total intensity of 37 J/cm^2^ and with a distance of 7 cm from the lesion to the light source. Following PDT, a cryostick constructed from solid CO_2_ was applied to the lesion twice for 5 sec (Fig. 1B). Images of all the lesions were obtained at baseline and then at 3 and 6 months. A skin biopsy from the lesion was obtained to evaluate the response and to histologically confirm complete healing.

## Results

**Figure 2:**
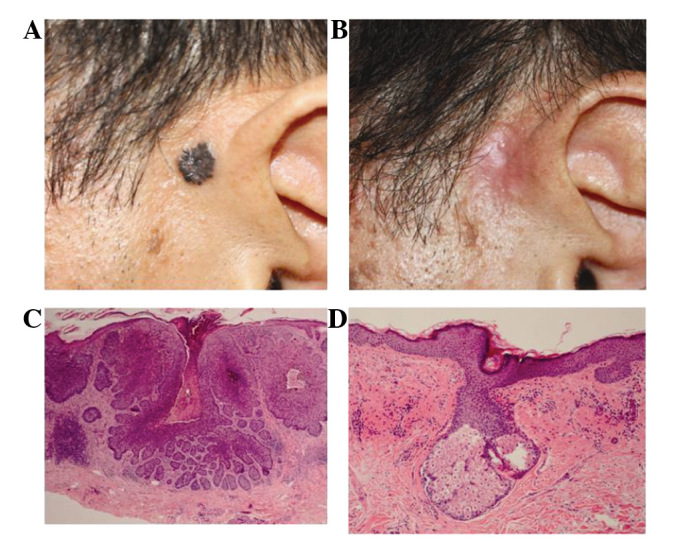
Nodular BCC lesion on the left temple of patient 5. Clinical images (A) prior to and (B) following combined PDT treatment. Histological images (C) prior to and (D) following treatment. BCC, basal cell carcinoma; PDT, photodynamic therapy.

**Figure 3:**
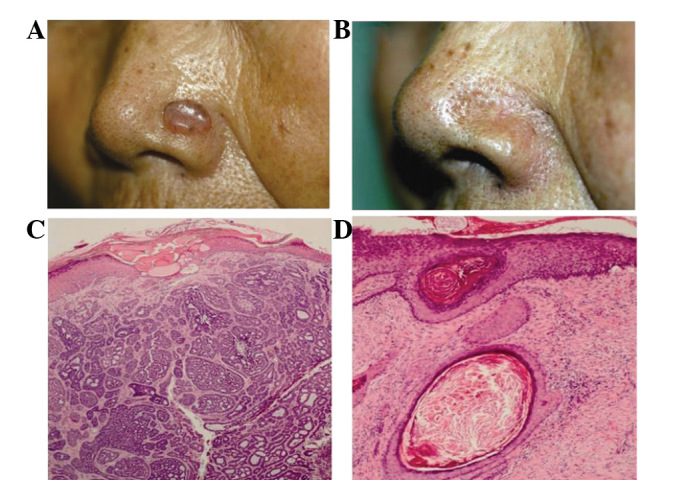
Nodular BCC lesion on the nose of patient 3. Clinical images (A) prior to and (B) following combined PDT treatment. Histological images (C) prior to and (D) following treatment. BCC, basal cell carcinoma; PDT, photodynamic therapy.

**Figure 4:**
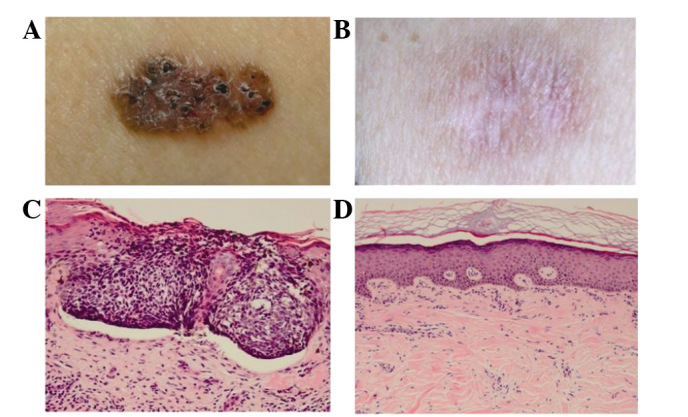
Superficial BCC lesion on the right thigh of patient 1. Clinical images (A) prior to and (B) following combined PDT treatment. Histological images (C) prior to and (D) following treatment. BCC, basal cell carcinoma; PDT, photodynamic therapy.

The mean age of the patients included in the study was 68 years (range, 57–78 years). A total of 6 lesions were on the face (Figs. 2 and 3), with 1 on the thigh (Fig. 4) and 1 on the arm. The histological subtypes of the BCC that were examined were varied, with 1 superficial, 6 nodular and 1 infiltrative (Table I). With regard to the tumor depth, 4 lesions were ≤2 mm deep and 4 lesions were >2 mm deep. The mean follow-up period was 22 months (range, 6–30 months). During the follow-up period, a total of 8 patients demonstrated a complete response following treatment with CO_2_ laser ablation in combination with PDT, followed by modified cryotherapy. In addition, no recurrences of disease were observed during this period (Figs 2 and 3). In total, 4 of the patients received 1 treatment session and 4 patients received 2 treatment sessions. Certain patients experienced a mild burning sensation during cryotherapy and the lesions exhibited post-inflammatory hyperpigmentation with minimal scarring (Fig. 4).

## Discussion

BCC is the most common type of skin cancer in humans. Of all BCCs, 85% occur in the head and neck region (4), therefore, the cosmetic outcome is a particularly important issue in the treatment of BCC. PDT is an effective option in the treatment of BCC due to its high cure rate and satisfactory cosmetic outcome (5). However, the efficacy of PDT monotherapy is limited in the treatment of deeper lesions (>2 mm depth) and the more aggressive subtypes of BCC (3). Therefore, deep curettage provides a favorable clinical and cosmetic short-term outcome following PDT due to the reduction in lesion thickness (6). Thus, further treatment modalities are required in addition to PDT for the treatment of the nodular and aggressive subtypes of BCC. For example, Whitaker *et al* reported that a combination of CO_2_ laser ablation and PDT plays a synergistic role in the treatment of nodular BCC (7).

In the present study, the data demonstrated that a combination of CO_2_ laser ablation and topical MAL PDT, followed by modified cryotherapy provided an effective method for the treatment of nodular and superficial BCC. Patients diagnosed with nodular BCC were completely healed with treated lesions exhibiting minimal hyperpigmentation and no scarring. This combined therapy was generally well tolerated in the patients, with the exception of certain patients who experienced a transient burning pain and erythema.

In the topical treatment of the deep, nodular and infiltrative subtypes of BCC using PDT, the delivery of sufficient photosensitizer and light to the full depth of the lesion is critical. The usage of CO_2_ laser ablation is beneficial since it removes the upper component of the BCC, thus potentially enhancing the penetrating depth of PDT and maximizing the efficacy of the treatment.

Conventional cryotherapy following PDT additionally kills the remaining cancer cells by interrupting vital metabolic cycles, destabilizing cell membranes, creating an adverse hyperosmolar environment and forming water crystals (8). In the present study, a solid cryostick was applied to the PDT lesion twice for 5 sec. The benefits of this modified form of cryotherapy include its ability to trigger a specific T lymphocyte response and to increase the activity of natural killer cells by releasing tumor antigens and inflammatory mediators. Thus, the application of a cryostick led to immunomodulation rather than the death of the tumor cells. Due to these synergistic effects, the present study demonstrated that this combination treatment was efficacious in the treatment of nodular and infiltrative subtypes of BCC, even when the tumor depth was >2 mm.

To the best of our knowledge, this is the first study to report the successful treatment of nodular BCC with a combination of CO_2_ laser ablation and PDT, followed by modified cryotherapy. We suggest that CO_2_ laser ablation in combination with PDT, followed by modified cryotherapy is a potential treatment option for nodular BCC.

## Figures and Tables

**Table I:** Characteristics of the patients treated with carbon dioxide laser ablation in combination with photodynamic therapy, followed by modified cryotherapy for BCC.

Case no.	Diagnosis	Gender	Age (years)	Location	Depth (mm)	No. of treatment sessions	Follow up (months)	Cosmetic outcome
1	Superficial BCC	F	63	Left thigh	0.55	1	30	PIH
2	Nodular BCC	F	78	Nose	1.12	1	29	Good
3	Nodular BCC	F	67	Nose	3.75	1	30	PIH
4	Nodular BCC	F	77	Right medial canthus	1.45	1	29	Good
5	Nodular BCC	M	64	Left temple	1.63	2	19	PIH
6	Nodular BCC	F	57	Right upper lip	2.54	2	16	Good
7	Nodular BCC	F	79	Left cheek	2.12	2	6	Good
8	Infiltrative BCC	M	61	Right arm	2.34	2	28	Good

PIH, post-inflammatoy hyperpigmentation; BCC, basal cell carcinoma; F, female; M, male.

## References

[1] Arits AH, Schlangen MH, Nelemans PJ, Kelleners-Smeets NW (2011). Trends in the incidence of basal cell carcinoma by histopathological subtype. J Eur Acad Dermatol Venereol.

[2] Szeimies RM, Ibbotson S, Murrell DF (2008). A clinical study comparing methyl aminolevulinate photodynamic therapy and surgery in small superficial basal cell carcinoma (8–20 mm), with a 12-month follow-up. J Eur Acad Dermatol Venereol.

[3] Fantini F, Greco A, Del Giovane C (2011). Photodynamic therapy for basal cell carcinoma: clinical and pathological determinants of response. J Eur Acad Dermatol Venereol.

[4] Miller SJ (1991). Biology of basal cell carcinoma (Part I). J Am Acad Dermatol.

[5] Rhodes LE, de Rie M, Enström Y (2004). Photodynamic therapy using topical methyl aminolevulinate vs surgery for nodular basal cell carcinoma: results of a multicenter randomized prospective trial. Arch Dermatol.

[6] Christensen E, Mørk C, Foss OA (2011). Pre-treatment deep curettage can significantly reduce tumour thickness in thick Basal cell carcinoma while maintaining a favourable cosmetic outcome when used in combination with topical photodynamic therapy. J Skin Cancer.

[7] Whitaker IS, Shokrollahi K, James W, Mishra A, Lohana P, Murison MC (2007). Combined CO(2) laser with photodynamic therapy for the treatment of nodular basal cell carcinomas. Ann Plast Surg.

[8] Messeguer F, Serra-Guillen C, Echeverria B (2012). A pilot study of clinical efficacy of imiquimod and cryotherapy for the treatment of basal cell carcinoma with incomplete response to imiquimod. J Eur Acad Dermatol Venereol.

